# Therapeutic role of corticosteroids in COVID-19: a systematic review of registered clinical trials

**DOI:** 10.1186/s43094-021-00217-3

**Published:** 2021-03-17

**Authors:** Reshma Raju, Prajith V., Pratheeksha Sojan Biatris, Sam Johnson Udaya Chander J.

**Affiliations:** College of Pharmacy, Sri Ramakrishna Institute of Paramedical Sciences, Coimbatore, Tamil Nadu India

**Keywords:** Methylprednisolone, Hydrocortisone, Steroids, COVID-19, SARS-CoV2, Clinical trial registry

## Abstract

**Background:**

In March 2020, the World Health Organization declared the coronavirus disease 2019 as a global pandemic. Though antiviral drugs and antimalarial drugs are considered treatment options for treating coronavirus disease 2019 (COVID-19), no specific antivirals are currently available for its treatment. Efficient use of drug discovery approaches including repurposing or repositioning of drugs used in the treatment of severe acute respiratory syndrome coronavirus (SARS-CoV) and the Middle East respiratory syndrome coronavirus (MERS-CoV) is considered recently. The widespread application of corticosteroid therapy in COVID-19 should be backed with careful documented pragmatic research of its use in this context.

**Main body:**

This article aims to analyze various trials registered across the globe providing an overall picture of the use of corticosteroids in the treatment of COVID-19. An extensive search was conducted on the clinical trial registries around the world to identify all the trials reporting information regarding the use of corticosteroids in COVID-19. Our initial search returned 231 trials, out of which 60 trials were finally included in the analysis. Fifty-six studies were interventional trials, and all the trials had clearly defined primary and secondary outcomes of interest, of which only 11 trials had evaluation of respiratory rate as one of their outcomes.

**Conclusion:**

Few preliminary trial findings show promising results and recommend the use of methylprednisolone and dexamethasone in the severe form of the disease; however, there is insufficient data to prove its benefits over its risks. Routine use of corticosteroids should be favored only after a better insight is obtained, with the completion of these trials.

## Background

**Fig. 1:**
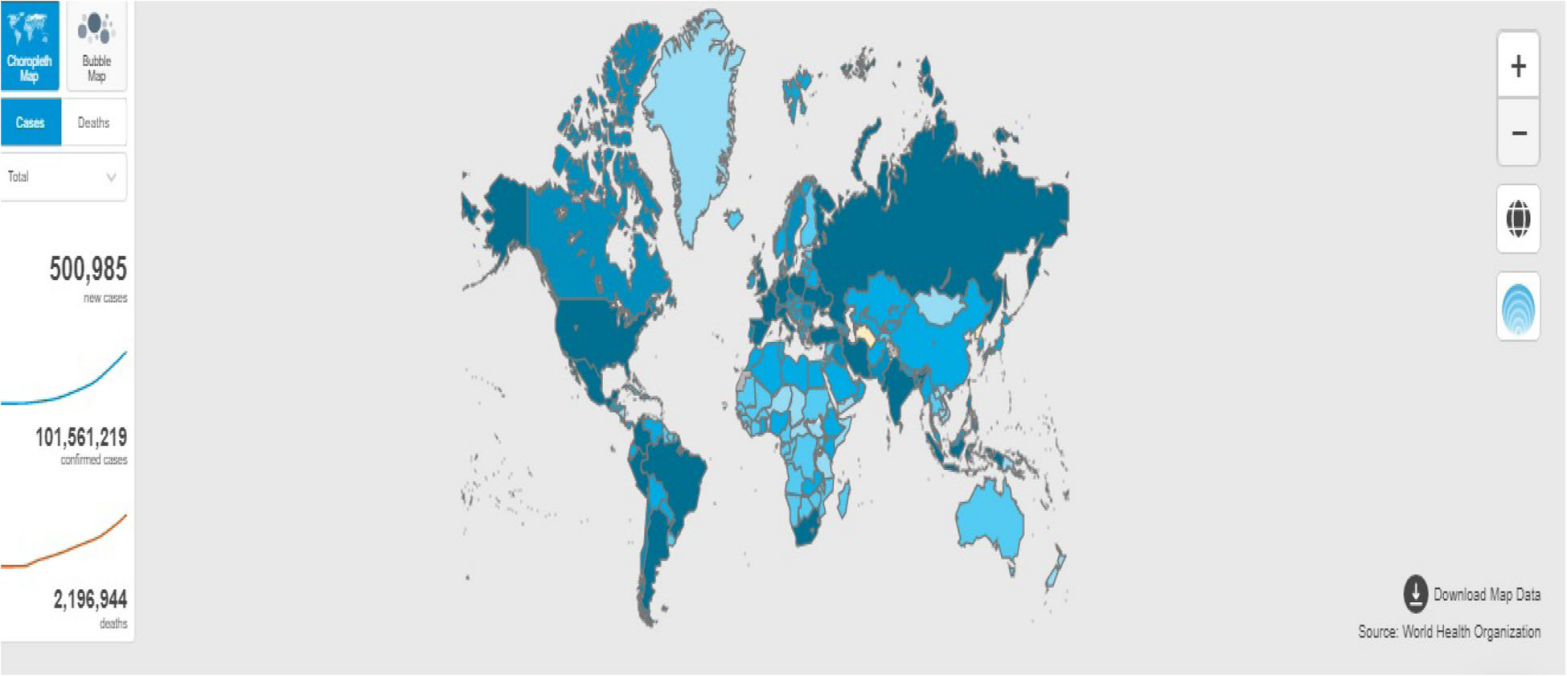
Global COVID-19 spread showing number of confirmed cases as of 30 January 2021 (Source: https://covid19.who.int/)

An outbreak of pneumonia caused by a new coronavirus spread in Wuhan province of China in December 2019. Sequencing of the sampling from patients with pneumonia revealed the viral genome phylogenetically closer to severe acute respiratory syndrome coronavirus (SARS-CoV) and the Middle East respiratory syndrome coronavirus (MERS-CoV) [1]. The Coronavirus Study Group named the causative agent severe acute respiratory syndrome coronavirus 2 (SARS-CoV-2), and the disease caused by this virus was named coronavirus disease 2019 (COVID-19 or 2019-nCoV) by the World Health Organization (WHO) [2, 3]. These viruses are enveloped, positive, single-stranded RNA viruses belonging to the family *Coronaviridae*, which can cause an array of symptoms including fever, dry cough, myalgia, fatigue, and dyspnea [4]. SARS-CoV-2 transmits from human-to-human by respiratory droplets caused by coughing or sneezing [5, 6]. The WHO declared COVID-19 as a Public Health Emergency of International Concern in January 2020 [7]. The infection has spread over to 216 countries (15,745,102 confirmed cases and 639,317 confirmed deaths) since its outbreak in November 2019 (as of 30 January 2021; Fig. 1).

Detection and diagnosis of this novel coronavirus mostly relied on molecular-based approaches such as nucleic acid testing, virus antigen, or serological antibody testing (against the N-protein of SARS-CoV) [8]. Treatment option includes antiviral drugs such as favipiravir, remdesivir, lopinavir, and ritonavir and antimalarial drugs such as chloroquine or hydroxychloroquine. Nevertheless, no vaccine or specific antiviral treatment recommended for COVID-19 is currently available [9]. The uncontrolled scenario of COVID-19 demands the use of effective drug discovery approaches for effective control of the disease [10–16]. Among these approaches, drug repurposing or drug repositioning is a time-effective way of treating a disease. One of the examples of successful application of drug discovery approach is drug repositioning of antivirals, and it has triggered a number of in vitro studies as well as clinical trials for a number of chemical molecules to evaluate their efficacy against COVID-19 [17–19]. Drug repurposing of corticosteroids has also been implemented recently as a part of a drug discovery approach. There are several studies reporting the use of corticosteroids in the treatment of severe coronavirus infections including COVID-19. The effectiveness of corticosteroids in some patients with SARS-CoV has resulted in a widespread application of this therapy in COVID-19, especially in patients in the ICU with severe infections, as these drugs prevent lung injury caused by severe community-acquired pneumonia (sCAP) due to their potential pharmacological effects on the suppression of exuberant and dysfunctional systematic inflammation [20].

## Main body

### Corticosteroids and their therapeutic role

The Infectious Diseases Society of America (IDSA) guidelines strongly recommends the use of dexamethasone in critically ill patients to treat acute respiratory distress syndrome (ARDS) and systemic inflammation, backed by moderate evidence. Dexamethasone at a total daily dose of 6 mg IV or PO for 10 days (or until discharge) or alternative glucocorticoids like methylprednisolone 32 mg and prednisone 40 mg are suggested. The level of recommendation decreases with decreasing severity of the disease. In non-severe COVID-19, the use of glucocorticoids is not recommended as there is a dearth of solid evidence. Additionally, experiences from SARS and MERS show risk of worsening clinical status, delayed viral clearance, and other adverse events [21]. Currently, available data on safety and effectiveness of corticosteroids in this setting is very few and inconclusive [20, 22, 23]. The value of corticosteroids as a treatment option in patients with severe COVID-19 infection needs careful documented pragmatic research in this context. In order to obtain strong clinical evidence, several studies have been launched that were registered on various clinical trial registries across the globe. The detailed analysis of these trials will give an overall picture of the use of corticosteroids in the treatment of COVID-19 around the world. This will help to identify the lacunae to be filled with definitive clinical evidence in order to reposition corticosteroid for COVID-19 treatment. Therefore, this study aims to analyze various trials registered across the globe providing an overall picture of the use of corticosteroids in the treatment of COVID-19.

### Search strategy

An extensive search was conducted to identify all the trials reporting information regarding the use of corticosteroids in COVID-19. We searched the following clinical trial registries: Clinicaltrials.gov, Chinese Clinical Trial Registry (ChiCTR), Clinical Research Information Service (CRiS)–Republic of Korea, EU Clinical Trials Register, ISRCTN Registry, Iranian Registry of Clinical Trials (IRCT), German Clinical Trials Register (DRKS), Japan Primary Registries Network (JPRN), and Clinical Trial Registry–India. The search was run until 23 June 2020. In Clinicaltrials.gov, the following keywords were used for search: “(COVID-19 OR SARS-CoV-2 OR 2019-nCoV OR severe acute respiratory syndrome coronavirus 2 OR Wuhan coronavirus OR 2019 novel coronavirus OR novel coronavirus–infected Pneumonia) AND (“glucocorticoids” OR “steroids” OR “corticosteroids” OR “hydrocortisone” OR “prednisone” OR “methylprednisolone” OR “dexamethasone” OR “prednisolone”). A similar strategy was adapted for the other registries. We included the English language and interventional and non-interventional studies. No restrictions were placed on the dose or formulation of the intervention. All trials must have studied the safety and efficacy of steroids in COVID-19 care.

### Recovery of trials

**Fig. 2:**
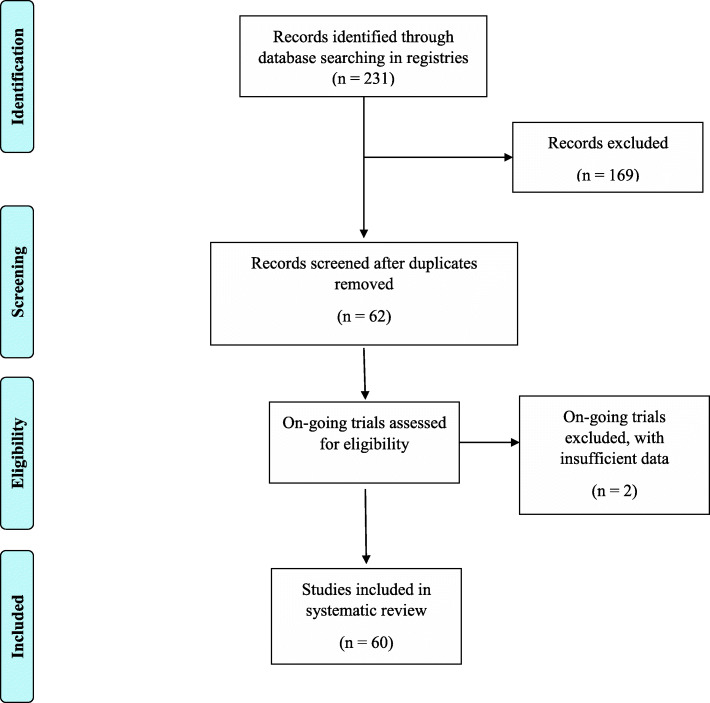
PRISMA flow diagram of reporting search results

Our initial search returned 231 trials, out of which 62 potentially relevant trials were identified. Potentially eligible trials were identified by three authors by screening titles and study description. All eligible trials were then assessed independently by three authors, and potentially relevant trials were selected in accordance with the predefined inclusion criteria. Any disagreement was reviewed and resolved by a fourth independent reviewer. Authors of individual trials were contacted if necessary. After a careful review of the study description, out of 62 articles, 2 trials did not satisfy the inclusion criteria and were excluded from the analysis. Finally, data from 60 trials were included in the final review and synthesis of results. This is shown in Fig. 2.

### Data abstraction and study appraisal

We extracted the following general data from each study: trial number, title, origin (country) of study, intervention, treatment arms, doses, mean age of participants, stage of COVID–19, expected start and end date of trial, primary outcomes of the study, blinding, randomization, and study design.

### Scrutiny of trials

Our initial search of the clinical trial registries resulted in 231 trials, of which 167 trials did not satisfy the inclusion criteria and three trials did not have complete data, and after removing the duplicates, 60 trials were included in the final analysis. Thus, 60 trials with 31,732 patients were included in this systematic review. The included trials were classified into trials that included only steroid therapy and those that included steroids in addition to other standard treatment as shown in Table 1.

**Table 1:** General characteristics of the included trials

Trial identifier	Country	Number of sites	Start date	Expected completion date	Stage of COVID-19	Outcome measures
NCT04425863	Argentina	Single	May 2020	July 2020	Severe acute respiratory syndrome	1. Illness development 2. Reduction of ICU admission 3. Mortality rate
NCT04395105	Argentina	Multi	May 2020	January 2021	Respiratory distress syndrome	Ventilator-free days at 28 days
NCT02735707	Australia, Belgium, Canada, Croatia, Germany, Hungary, Ireland, Netherlands, New Zealand, Portugal, Romania, Spain, UK	Multi	April 2020	December 2023	Pneumonia	1. All-cause mortality 2. Days alive and outside of ICU
NCT04343729	Brazil	Single	April 2020	September 2020	Severe acute respiratory syndrome (SARS)	Mortality rate
NCT04327401	Brazil	Multi	April 2020	August 2020	Moderate/severe ARDS	Ventilator-free days
NCT04377503	Brazil	Not available	May 2020	November 2020	Cytokine release syndrome	Patient clinical status 15 days after randomization
NCT04374474	Canada	Single	January 2021	March 2022	Not defined	1. Change from Baseline Snap and Sniff Threshold Test at 3 months 2. Score from the Snap and Sniff Olfactory Test results 3. Change from baseline Smell Identification Test (SIT) at 3 months 4. Score from the Smell Identification test results. 5. Change from Baseline Snap and Sniff Threshold Test at 6 months 6. Score from the Snap and Sniff Olfactory Test results 7. Change from baseline Smell Identification Test (SIT) at 6 months
NCT04263402	China	Not available	February 2020	July 2020	Severe pneumonia	1. Rate of disease remission 2. Rate and time of entering the critical stage
NCT04244591	China	Completed	January 2020	April 2020	Severe acute respiratory failure	Murray lung injury score
NCT04273321	China	Completed	February 2020	April 2020	Pneumonia	The incidence of treatment failure in 14 days
ChiCTR2000029386	China	Single	January 2020	January 2021	Pneumonia	SOFA score
ChiCTR2000029656	China	Single	February 2020	April 2020	Pneumonia	ECG, chest imaging, complications, vital signs, and NEWS2 score
ChiCTR2000030481	China	Multi	January 2020	April 2020	Pneumonia	The time of duration of COVID-19 nucleic acid RT-PCR test results of respiratory specimens (such as throat swabs) or blood specimens change to negative
NCT04348305	Denmark	Multi	April 2020	December 2021	COVID-19 hypoxia	Days alive without life support at day 28
2020-001395-15	Denmark	Multi	April 2020	Not available	Severe hypoxia	1. Days alive without life support (i.e., invasive mechanical ventilation, circulatory support or renal replacement therapy) from randomization to day 28).
NCT04331054	France	Multi	April 2020	July 2020	COVID-19 infection	Time (in days) to clinical improvement within 30 days after randomization
NCT04361474	France	Multi	May 2020	May 2021	Hyposmia	Patient with more than 2 points on the ODORATEST
NCT04359511	France	Not available	June 2020	December 2020	Pneumonia	Clinical improvement defined by the improvement of 2 points on a 7-category ordinal scale, at 14 days
NCT04347980	France	Multi	April 2020	August 2020	Acute respiratory distress syndrome (ARDS)	28-day mortality
NCT04344730	France	Single	April 2020	December 2020	Pneumonia	1. Time-to-death from all causes within the first 60 days after randomization 2. Time to need for mechanical ventilation
NCT04344288	France	Multi	April 2020	November 2020	Pneumonia	Respiratory indication for transfer to intensive care unit evaluated by a SpO2 < 90%
NCT04331470	Iran	Single	April 2020	May 2020	Not defined	Clear chest CT scan and PCR test
IRCT20200204046369N1	Iran	Multi	Not available	Not available	Not defined	PAO2/FiO2 through ABG method
IRCT20151227025726N17	Iran	Single	Not available	Not available	ARDS	1. Daily need for invasive mechanical ventilation 2. Death at the end of the study
IRCT20120225009124N4	Iran	Single	Not available	Not available	Not defined	Improvement in SpO2 measured by pulse oximeter
IRCT20200406046963N1	Iran	Single	Not available	Not available	ARDS( acute respiratory distress syndrome)	1. Mortality rate after 60 days 2. Blood O2 saturation measurement 3. Need for oxygen therapy
IRCT20200404046947N1	Iran	Multi	Not available	Not available	Not defined	1. Findings on the CT scan 2. Mortality rate 3. O2 saturation levels 4. Need an oxygen therapy at day 3 and discharge time
IRCT20081027001411N3	Iran	Multi	Not available	Not available	ARDS	1. Findings on the CT scan 2. Mortality rate 3. O2 saturation levels 4. Need an oxygen therapy at day 3 and discharge time
IRCT20120215009014N354	Iran	Single	Not available	Not available	Mild-to-moderate acute respiratory distress syndrome	1. Need to mechanical ventilation 2. The patient’s clinical status 3. Mortality rate
IRCT20080901001165N52	Iran	Single	Not available	Not available	Moderate to severe pneumonia	Need to receive ICU service
NCT04323592	Italy	Single	March 2020	May 2020	Acute respiratory distress syndrome	1. Admission to ICU and need for Invasive mechanical ventilation 2. In-hospital death within 28 days 3. Endotracheal intubation
KCT0005105	Korea	Multi	April 2020	September 2020	Mild	Rate of SARS-CoV-2 eradication at day 14 from study enrollment
IRCT20200318046812N2	Iran	Multi	Not available	Not available	Not defined	Admission to intensive care unit
NCT04345445	Malaysia	Single	April 2020	October 2020	Pneumonia	1. The proportion of patients requiring mechanical ventilation 2. Mean days in ventilation
NCT04360876	Not available	Single	May 2020	December 2020	ARDS	Ventilator-free days (VFD) at day 28
NCT04366115	Not available	Not available	June 2020	June 2023	Not defined	1. Dose-limiting toxicities 2. 28-day all-cause mortality for phases 1 and 2
NCT04435795	Not available	Not available	June 2020	March 2021	Not defined	Improvement in dyspnea at day 7
NCT04355247	Puerto Rico	Multi	April 2020	April 2021	High-risk COVID-19	1. Clinical complete response criteria 2. Need for ventilatory support 3. O2 Saturation of >/= 93% by day 14 of therapy 4. Mortality at day 28 5. Findings on CT chest on day 28
NCT04438980	Spain	Multi	May 2020	February 2021	Pneumonia	1. Proportion of patients developing treatment failure 2. Need for mechanical ventilation 3. Decrease in SpO2 < 90% (in ambient air) or PaO2 < 60 mmHg (in ambient air) or PaO2FiO2 < 300 mmHg
NCT04394182	Spain	Multi	April 2020	April 2021	Pneumonia	Oxygen saturation at day 2
NCT04380818	Spain	Multi	June 2020	July 2021	Pneumonia	Efficacy of low-dose pulmonary irradiation assessed by change in PAFiO2 by 20%
NCT04355637	Spain	Multi	April 2020	October 2020	Pneumonia	Proportion of patients developing treatment failure
NCT04341038	Spain	Single	April 2020	June 2020	Severe lung injury secondary to COVID-19	Time to reach clinical stability
NCT04329650	Spain	Multi	April 2020	May 2020	Pneumonia	Proportion of patients requiring ICU admission at any time within the study period
NCT04325061	Spain	Multi	April 2020	October 2020	ARDS	60-day mortality
2020-001827-15	Spain	Single	Not available	Not available	Pneumonia	1. Proportion of patients with treatment failure up to 14 days after randomization 2. Mortality rate 3. ICU admission 4. Number of patients requiring mechanical ventilation 5. Clinical deterioration/worsening, defined as decrease in SpO2 below 90% or PaO2 below 60 mmHg in ambient air + radiological progression.
2020-001622-64	Spain	Single	April 2020	Not available	Not defined	1. Measurement of O2 saturation and/or blood gas, findings on chest x-ray, CBC, including inflammatory markers and blood biometrics, and ECG 2. 30-day ICU admission and hospital stay 3. Outbreaks of steroid-related psychosis
2020-001934-37	Spain	Multi	May 2020	Not available	Not defined	1. Mortality rate 2. Number of days of ICU stay 3. Number of patients requiring non-invasive ventilation (NIV)
2020-001413-20	Spain	Single	April 2020	Not available	Pneumonia	Proportion of patients requiring ICU admission at any time within the study period
2020-001445-39	Spain	Single	March 2020	Not available	Pneumonia	Time (days) to clinical stability after initiation of trial treatment for severe pneumonia secondary to COVID-19 and elevated inflammatory parameters
2020-001307-16	Spain	Single	April 2020	Not available	ARDS	Death from any cause in the first 28 days after randomization
NCT04381364	Sweden	Multi	May 2020	December 2020	Pneumonia	Duration of supplemental oxygen therapy
NCT04416399	UK	Not available	June 2020	December 2020	Early infection	Emergency department visit related to COVID-19
NCT04381936	UK	Multi	March 2020	June 2021	SARS	All-cause mortality
NCT04411667	USA	Multi	April 2020	November 2020	Not defined	Number of subjects requiring mechanical ventilation
NCT04377711	USA	Multi	June 2020	December 2020	Symptomatic COVID-19 infection	Percentage hospital admission or death by day 30
NCT04349410	USA	Not available	April 2020	November 2020	Pneumonia	1. Improvement in FMTVDM measurement with nuclear imaging 2. Ventilator status 3. Extubation status 4. Survival status in 30 days
NCT04193878	USA	Multi	June 2020	June 2024	Pneumonia, acute respiratory failure	Number of patients with acute respiratory failure (ARF) within 10 days of randomization
NCT03852537	USA	Single	December 2019	July 2022	Pneumonia	1. Feasibility of the timely initiation of corticosteroids and implementation of biomarker-titrated corticosteroid dosing 2. Percentage of eligible patients adhered to the timely initiation within 30 days
NCT04374071	USA	Completed	March 2020	April 2020	Pneumonia	1. Number of patients transferred to ICU is each of the group 2. Number of patients requiring mechanical ventilation 3. Mortality rate

### Type of trials

Among the included trials, 57 trials were quantitative studies and the remaining three trials were qualitative studies, i.e., non-interventional studies, as shown in the Table 2.

**Table 2:** Methodological quality of included trials

Trial identifier	Estimated sample size	Allocation (randomized/non-randomized)	Blinding/masking	Study design
NCT04438980	72	Randomized	Double	Interventional
NCT04435795	454	Randomized	Triple	Interventional
NCT04425863	10	Not available	Not available	Non-interventional, prospective cohort
NCT04416399	478	Randomized	Open label	Interventional
NCT04411667	40	Randomized	Open label	Interventional
NCT04395105	284	Randomized	Open label	Interventional
NCT04394182	15	Not available	Open label	Interventional
NCT04381936	12000	Randomized	Open label	Interventional
NCT04348305	1000	Randomized	Quadruple	Interventional
NCT04331054	436	Randomized	Open label	Interventional
NCT04360876	90	Randomized	Double	Interventional
NCT04355247	20	Not available	Open label	Interventional
NCT04381364	446	Randomized	Open label	Interventional
NCT04380818	106	Non-randomized	Open label	Interventional
NCT04377711	400	Randomized	Double	Interventional
NCT04377503	40	Randomized	Open label	Interventional
NCT04374474	75	Randomized	Open label	Interventional
NCT04366115	126	Randomized	Open label	Interventional
NCT04361474	120	Randomized	Single	Interventional
NCT04359511	210	Randomized	Single	Interventional
NCT04355637	300	Randomized	Open label	Interventional
NCT04349410	500	Randomized	Single	Interventional
NCT04347980	122	Randomized	Single	Interventional
NCT04263402	100	Randomized	Single	Interventional
NCT04193878	600	Randomized	Triple	Interventional
NCT03852537	90	Randomized	Double	Interventional
NCT02735707	7100	Randomized	Open label	Randomized, multifactorial trial
NCT04345445	310	Randomized	Open label	Interventional
NCT04344730	550	Randomized	Quadruple	Interventional
NCT04344288	304	Randomized	Open label	Interventional
NCT04343729	425	Randomized	Quadruple	Interventional
NCT04341038	84	Randomized	Single	Interventional
NCT04331470	30	Randomized	Double	Interventional
KCT0005105	141	Randomized	Open label	Interventional
NCT04329650	200	Randomized	Open label	Interventional
NCT04327401	350	Randomized	Open label	Interventional
NCT04325061	200	Randomized	Open label	Interventional
NCT04323592	173	Non-randomized	Open label	Non-interventional, prospective cohort
NCT04374071	250	Non-randomized	Not available	Non-interventional, retrospective cohort
NCT04244591	80	Randomized	Open label	Interventional
NCT04273321	86	Randomized	Open label	Interventional
IRCT20080901001165N52	50	Randomized	Open label	Interventional
IRCT20200406046963N1	40	Randomized	Open label	Interventional
IRCT20200404046947N1	68	Randomized	Single	Interventional
IRCT20081027001411N3	60	Randomized	Single	Interventional
IRCT20120215009014N354	81	Randomized	Double	Interventional
IRCT20200204046369N1	48	Non-randomized	Open label	Interventional
IRCT20200318046812N2	906	Randomized	Open label	Interventional
IRCT20151227025726N17	48	Randomized	Open label	Interventional
IRCT20120225009124N4	105	Randomized	Open label	Interventional
ChiCTR2000029386	24	Randomized	Not available	Interventional
ChiCTR2000029656	50	Randomized	Open label	Interventional
ChiCTR2000030481	75	Randomized (static)	Not available	Interventional
2020-001827-15	72	Randomized	Double	Interventional
2020-001307-16	104	Randomized	Open label	Interventional
2020-001395-15	1000	Randomized	Double	Interventional
2020-001622-64	200	Randomized	Open label	Interventional
2020-001934-37	200	Randomized	Open label	Interventional
2020-001413-20	100	Randomized	Open label	Interventional
2020-001445-39	84	Randomized	Open label	Interventional

### Heterogeneity of trials

All 60 trials included were heterogenous in that they had various inclusion and exclusion criteria and different treatment protocols for the treatment of various stages of COVID-19. The most common stage of COVID-19 among these trials is pneumonia, which is shown in Table 1.

### Methodological quality of the trials

Among the 60 trials, 54 were randomized. It was unclear how randomization was carried out in three of the trials. Among 54 randomized trials, only 21 trials were blinded, of which 8 were single blinded, 8 were double blinded, 2 were triple blinded, and 3 were quadruple blinded, as shown in Table 2.

### Steroid treatment

Regarding the steroid treatment, the most common steroid used is methylprednisolone (used in 28 trials) at various dosages depending on the age of the patients. Maximum loading dose of methylprednisolone used is 500 mg IV infusion over 1h in a trial (IRCT20080901001165N52). Steroids were given from a minimum of 3 days to a maximum of 21 days. Other steroids used are budesonide, ciclesonide, dexamethasone, formoterol, prednisolone, prednisone, and hydrocortisone. In 10 trials, the dose of the steroids used was unclear, and in one trial (ChiCTR2000030481), the treatment regimen was not mentioned. This is shown in Table 3.

**Table 3:** Steroid treatment in patients with COVID-19

Trial identifier	Title	Interventions	Dose	Age (in years)
NCT04425863	Evaluation of ivermectin, aspirin, dexamethasone, and enoxaparin as treatment of Covid19	Ivermectin; aspirin; dexamethasone; enoxaparin	Dexamethasone 4 mg/day IV	≥ 5
NCT04395105	Dexamethasone versus usual care for the treatment of COVID-19 related ARDS: a multicenter and randomized open-label clinical trial	Dexamethasone	Dexamethasone 16 mg IV OD from days 1 to 5 and 8 mg from days 6 to 10	≥ 18
NCT02735707	Randomized, embedded, multifactorial adaptive platform trial for community-acquired pneumonia	1. Fixed-duration Hydrocortisone 2. Shock-dependent hydrocortisone 3. Ceftriaxone 4. Moxifloxacin or Levofloxacin 5. Piperacillin-tazobactam 6. Ceftaroline 7. Amoxicillin-clavulanate 8. Macrolide administered for 3–5 days 9. Macrolide administered for up to 14 days 10. Five days of oseltamivir 11. Ten days of oseltamivir 12. Lopinavir/ritonavir 12. Hydroxychloroquine 13. Hydroxychloroquine + lopinavir/ritonavir 14. Interferon-β1a 15. Anakinra 16. Fixed-duration higher dose hydrocortisone 17. Tocilizumab 18.Sarilumab	1. Fixed-duration hydrocortisone 50 mg IV q 6 h × 7 days 2. Shock-dependant hydrocortisone 50 mg IV q 6 h while in septic shock 3. Fixed-duration higher dose hydrocortisone—100 mg IV every 6 h × 7 days	> 18
NCT04377503	Comparison of the efficacy and safety of tocilizumab versus methylprednisolone in the cytokine release syndrome of patients with COVID-19. A prospective randomized controlled phase II trial	1. Tocilizumab 180 mg/ml 2. Methylprednisolone sodium succinate	Methylprednisolone sodium succinate 1.5 mg/kg/day BD × 7 days followed by 1 mg/kg/day × 7 days, finally 0.5 mg/kg/day × 21 days	≥ 18
NCT04343729	Methylprednisolone in the treatment of patients with signs of severe acute respiratory syndrome in Covid-19 (MetCOVID)	Methylprednisolone sodium succinate	Methylprednisolone sodium succinate 0.5 mg/kg	≥ 18
NCT04327401	COVID-19-associated ARDS treated with dexamethasone: Alliance Covid-19 Brasil III (CoDEX)	Dexamethasone	Dexamethasone 20 mg IV 1/day × 5 days followed by 10 mg IV 1/day × 5 days	≥ 18
NCT04374474	Olfactory retraining therapy and budesonide nasal rinse for anosmia treatment in patients post-CoVID 19. A randomized controlled trial	1. Corticosteroid nasal irrigation 2. Smell household items; olfactory retraining	Budesonide 240 ml nasal irrigation with Pulmicort Respules (0.5 mg) across both nose sides	≥ 18
NCT04263402	An open, prospective/retrospective, randomized controlled cohort study to compare the efficacy of different hormone doses in the treatment of 2019-nCoV severe pneumonia	Methylprednisolone	1. Methylprednisolone < 40 mg/day IV drip × 7 days 2. Methylprednisolone 40 to 80 mg/day IV drip × 7 days	≥ 18
NCT04244591	Glucocorticoid therapy for critically ill patients with severe acute respiratory infections caused by COVID-19: a prospective, randomized controlled trial	Methylprednisolone therapy. Others: standard care	Methylprednisolone 40 mg q. 12 h × 5 days	≥ 18
NCT04273321	Efficacy and safety of corticosteroids in COVID-19: a prospective randomized controlled trials	Methylprednisolone	Accord with the clinical diagnosis and/or etiological diagnosis diagnostic criteria	18–75
ChiCTR2000029386	Effectiveness of glucocorticoid therapy in patients with severe novel coronavirus pneumonia: a randomized controlled trial	Methylprednisolone and intravenous injection	Methylprednisolone 1–2 mg/kg/day IV × 3 days	≥ 18
ChiCTR2000029656	A randomized, open-label study to evaluate the efficacy and safety of low-dose corticosteroids in hospitalized patients with novel coronavirus pneumonia (COVID-19)	Methylprednisolone	Not available	≥ 18
ChiCTR2000030481	The clinical value of corticosteroid therapy timing in the treatment of novel coronavirus pneumonia (COVID-19): a prospective randomized controlled trial	Not mentioned	Not available	≥ 18
NCT04348305	Low-dose hydrocortisone in patients with COVID-19 and severe hypoxia - the COVID STEROID Trial	Hydrocortisone	Hydrocortisone continuous infusion: 200 mg q 24 h bolus injections 50 mg (10 ml) every 6 h × 7 days	≥ 18
NCT04331054	Protective role of inhaled steroids for Covid-19 infection	1. Usual practice 2. Usual practice + Symbicort Rapihaler	Symbicort (budesonide, formoterol) 200/6 μg, 2 puffs bid × 30 days	18–75
NCT04361474	A randomized controlled trial evaluating the efficacy of local budesonide therapy in the management of hyposmia in COVID-19 patients without signs of severity	1. Budesonide nasal spray 2. Physiological serum	Budesonide 1 mg/2 ml diluted in 250 ml of physiological saline 3 syringes of 20 ml in each nasal cavity BD × 30 days	≥ 18
NCT04359511	Efficacy and safety of corticosteroids in oxygen-dependent patients with COVID-19 pneumonia in Grand Ouest Interregion France	1. Prednisone 2. Hydrocortisone	Prednisone 0.7 mg/kg/day PO OD × 10 days or hydrocortisone hemisuccinate 3.5 mg/kg/day continuous infusion × 10 days	≥ 18
NCT04347980	Dexamethasone combined with hydroxychloroquine compared to hydroxychloroquine alone for treatment of severe acute respiratory distress syndrome induced by coronavirus disease 19 (COVID-19): a multicentre, randomised controlled trial	1. Dexamethasone and hydroxychloroquine 2. Hydroxychloroquine	Dexamethasone 20 mg IV OD for 15 min × 5 days followed by 10 mg OD × 5 days	≥ 18
NCT04344730	Dexamethasone and oxygen support strategies in ICU patients with Covid-19 pneumonia (COVIDICUS)	Dexamethasone injection + conventional oxygen	Dexamethasone 20 mg/5 ml IV	18–80
NCT04344288	Corticosteroids during Covid-19 viral pneumonia related to SARS-Cov-2 infection (CORTI-Covid)	Prednisone	Prednisone 0.75 mg/kg/day × 5 days then 20 mg/day × 5 more days	≥ 18
NCT04331054	Protective role of inhaled steroids for Covid-19 infection	1. Usual practice 2. Usual practice + Symbicort Rapihaler	Symbicort (budesonide, formoterol) 200/6 μg 2 puffs bid × 30 days	18–75
NCT04331470	Evaluation of efficacy of levamisole and formoterol + budesonide in treatment of COVID-19	1. Levamisole pill + budesonide + formoterol inhaler/lopinavir/ritonavir + hydroxychloroquine 2. Lopinavir/ritonavir + hydoxychloroquine	Budesonide + formoterol inhalation 1–2 puffs q 12 h	15–100
IRCT20080901001165N52	Investigating the efficacy of high dose of glucocorticoid in patients with moderate to severe pneumonia related to COVID-19	Methylprednisolone and prednisolone	*Day 1*: Amp. methylprednisolone 500 mg IV infusion over 1 hour. *At days 2 and 3*: Amp. methylprednisolone 250 mg IV infusion over 1 h. *At days 4 and 5*: Amp. methylprednisolone 100 mg IV infusion over 1 h. Then, tab. prednisolone 25 mg PO daily until the day of discharge, then tab. prednisolone will gradually tapered off over 1 month	18–85
IRCT20200204046369N1	Evaluation of methylprednisolone administration as a therapeutic option in the 2019 novel coronavirus (COVID-19): a non-randomized controlled study	Methylprednisolone	Methylprednisolone 20 mg/day	≥ 18
IRCT20200318046812N2	Safety and efficacy of “Hydroxychloroquine + Azithromycin + naproxen + Prednisolone” and “Hydroxychloroquine + Azithromycin + naproxen” regimens in comparison with “Hydroxychloroquine + kaletra” on the need for intensive care unit treatment in patients with COVID-19; a randomized, multicenter, parallel	Hydroxychloroquine, azithromycin, naproxen , prednisolone	Prednisolone five 5 mg tablets a day × 5 days	16–100
IRCT20151227025726N17	Evolution of the efficacy and safety of Dexamethasone administration in patients with mild to moderate COVID-19 acute respiratory disease syndrome	Dexamethasone	Dexamethasone 20 mg IV days 1–5, then 10 mg days 6–10	≥ 18
IRCT20120225009124N4	Efficacy of different methods of administration of combination regimen including dexamethasone, IV-IG and interferon beta for treatment of patients with severe COVID-19: a randomized controlled trial	Dexamethasone, IV-IG and interferon beta	Not available	18–70
IRCT20200406046963N1	Evaluation of the efficacy and safety of methylprednisolone pulse therapy in treatment of COVID-19 patients with ARDS.	Methylprednisolone	Methylprednisolone 1000 mg for 3 days	18–90
IRCT20200404046947N1	Study of methylprednisolone effects on treatment and clinical symptoms and laboratory signs of Iranian COVID-19 patients: a clinical trial study	Methylprednisolone	Methylprednisolone 250 mg for 3 days	≥ 18
IRCT20081027001411N3	Study of prednisolone effects on treatment and clinical symptoms and laboratory signs of Iranian COVID-19 patients: a clinical trial study	Prednisolone	Prednisolone 0.5 mg/kg in three divided doses up to 30 mg per day for 5–7 days	≥ 18
IRCT20120215009014N354	Evaluating the effect of intravenous hydrocortisone, methylprednisolone, and dexamethasone in treatment of patients with moderate to severe acute respiratory distress syndrome caused by COVID-19: a double blind randomized clinical trial	Hydrocortisone, methylprednisolone, and dexamethasone	*Group 1*: Hydrocortisone 50 mg IV q. 6 h × 5 days *Group 2*: Methylprednisolone 40 mg IV q 12 h ×5 days *Group 3*: Dexamethasone IV 20 mg daily × 5 days	18–70
NCT04323592	Methylprednisolone for patients with COVID-19 severe acute respiratory syndrome (MP-C19)	Methylprednisolone and other standard care	Methylprednisolone 80 mg/kg IV bolus	18–80
KCT0005105	A trial of ciclesonide in adults with mild COVID-19	1. Ciclesonide (Alvesco®) 320 μg inhalation twice a day for 14 days 2. Ciclesonide (Alvesco®) 320 μg inhalation twice a day for 14 days + hydroxychloroquine 400 mg per day for 10 days	Ciclesonide (Alvesco®) 320 μg inhalation BD × 14 days	19–100
NCT04345445	Study to evaluate the efficacy and safety of tocilizumab versus corticosteroids in hospitalized COVID-19 patients with high risk of progression	1. Tocilizumab IV 2. Methylprednisolone IV	Methylprednisolone 120 mg/day for 3 days	18–80
NCT04435795	Ciclesonide clinical trial for COVID-19 treatment	Ciclesonide	Ciclesonide 600 μg BID inhaled with aerochamber + Nasal ciclesonide 200 μg DIE	≥ 18
NCT04360876	Targeted steroids for ARDS due to COVID-19 pneumonia: a pilot randomized clinical trial	1. Dexamethasone injection 2. Placebo	Dexamethasone 20 mg IV OD 5 days followed by 10 mg OD × 5 days	≥ 18
NCT04366115	A randomized, double-blind, placebo-controlled, phase 1/2 study evaluating AVM0703 in patients with COVID-19	1. AVM0703 2. Placebo 3. Hydrocortisone	1. AVM0703 (dexamethasone sodium phosphate) 10 mg/ml single IV infusion in NS over 1 hour 2. Hydrocortisone dose not available	≥ 18
NCT04355247	Prophylactic corticosteroid to prevent COVID-19 cytokine storm	Methylprednisolone 80 mg/ml injectable suspension	Methylprednisolone 80 mg IV bolus injection OD × 5 days	≥ 18
NCT04438980	Treatment of COVID-19 pneumonia with glucocorticoids. A randomized controlled trial	1. Methylprednisolone 2. Placebo	Methylprednisolone 120 mg/day IV infusion × 3 days	18–80
NCT04394182	Low doses of lung radiation therapy in cases of COVID-19 pneumonia: prospective multicentric study in radiation oncology centers	1. Ultra-low-dose radiotherapy 2. Ventilatory support with oxygen therapy 3. Lopinavir/ritonavir, hydroxychloroquine, azithromycin, piperacillin/tazobactam, Low molecular weight heparin, corticosteroid injection, tocilizumab	Methylprednisolone 250 mg × 3 boluses	18–120
NCT04380818	Low dose anti-inflammatory radiotherapy for the treatment of pneumonia by COVID-19: multi-central prospective study	1. Low-dose radiotherapy; hydroxychloroquine Sulfate 2. Ritonavir/lopinavir 3. Tocilizumab Injection (Actemra) 4. Azithromycin 5. Corticosteroid 6. Low molecular weight heparin; oxygen supply	Not available	18–99
NCT04355637	Treatment with inhaled corticosteroids in patients hospitalized because of COVID19 pneumonia	Inhaled budesonide	Not available	18–79
NCT04341038	Clinical trial to evaluate methylprednisolone pulses and tacrolimus in patients with COVID-19 lung injury (TACROVID)	1. Tacrolimus 2. Methylprednisolone	Methylprednisolone 120 mg daily × 3 days	Not available
NCT04329650	Efficacy and safety of siltuximab vs. corticosteroids in hospitalized patients with COVID-19 pneumonia	1. Siltuximab 2. Methylprednisolone	Methylprednisolone 250 mg/24 h	≥ 18
NCT04325061	Efficacy of dexamethasone treatment for patients with ARDS caused by COVID-19 (DEXA-COVID19)	Dexamethasone	Dexamethasone 20 mg/IV/daily × 5 days	> 18
2020-001827-15	Early treatment of pneumonia Covid-19 with glucocorticoids. randomized controlled clinical trial	Methylprednisolone and hydroxychloroquine	Not available	≥ 18
2020-001622-64	Outpatient treatment of Covid-19 with early pulmonary corticosteroids as an opportunity to modify the course of the disease	Prednisone	Not available	18–74
2020-001934-37	Use of corticosteroids in patients with SARS-CoV2 coronavirus infection (glucocovid) pragmatic trial inserted in real practice during a pandemic covid-19	Methylprednisolone	Not available	18–85
2020-001413-20	Phase 2, randomized, open-label study to compare the efficacy and safety of siltuximab vs. corticosteroids in hospitalized patients with COVID-19 pneumonia	Methylprednisolone and siltuximab	Not available	≥ 18
2020-001445-39	Pragmatic, controlled, open, single center, randomized, phase Ii clinical trial to evaluate methylprednisolone pulses and tacrolimus in hospitalized patients with severe pneumonia secondary to COVID-19.	1. Methylprednisolone 2. Tacrolimus	Not available	≥ 18
2020-001307-16	Efficacy and safety of corticoids in patients with adult respiratory distress syndrome (ARDS) secondary to COVID-19.	Methylprednisolone hemisuccinate	Not available	≥ 18
NCT04381364	Inhalation of ciclesonide for patients with COVID-19: a randomised open treatment study (HALT COVID-19)	Ciclesonide inhalation	Ciclesonide inhalation 320 μg BD × 14 days	18–84
NCT04416399	Use of high dose inhaled corticosteroids as treatment of early COVID-19 infection to prevent clinical deterioration and hospitalization	Budesonide dry powder inhaler	Budesonide 400 μg per inhalation, 2 inhalations twice a day × 28 days	> 18
NCT04381936	Randomized evaluation of COVID-19 therapy	1. Lopinavir-ritonavir 2. Dexamethasone/prednisolone 3. Hydroxychloroquine 4. Azithromycin 5. Biological: convalescent plasma 6. Tocilizumab	Dexamethasone 6 mg PO OD × 10 days	Child, adult, older adult
NCT04411667	Randomized open label study of standard of care plus intravenous immunoglobulin (IVIG) compared to standard of care alone in the treatment of COVID-19 infection	IVIG (Octagam) premedication and methylprednisolone	Methylprednisolone 40 mg IV push × 1 30–50 min before each IVIG infusion	≥ 18
NCT04377711	A phase 3, multicenter, randomized, double-blind, placebo-controlled study to assess the safety and efficacy of ciclesonide metered-dose inhaler in non-hospitalized patients 12 years of age and older with symptomatic COVID-19 infection	1. Ciclesonide 2. Placebo	Alvesco (ciclesonide) 320 μg b.i.d. × 30 days via pMDI	12–100
NCT04349410	The fleming [FMTVDM] directed CoVid-19 treatment protocol	1. Hydroxychloroquine, azithromycin 2. Hydroxychloroquine, doxycycline 3. Hydroxychloroquine, clindamycin 4. Hydroxychloroquine, clindamycin, primaquine—low dose 5. Hydroxychloroquine, clindamycin, primaquine—high dose 6. Remdesivir 7. Tocilizumab 8. Methylprednisolone 9. Interferon-Alpha2B 10: Losartan plus convalescent serum	Methylprednisolone 80 mg IV over 30 min b.i.d. × 7 days, then taper off	Child, adult, older adult
NCT04193878	Arrest respiratory failure from pneumonia (Arrest pneumonia)	1. Inhaled budesonide and formoterol 2. Inhaled placebo	Formoterol aerosolized—20 μg/2 ml) and budesonide—1.0 mg/2 ml q 12 h × 14 doses	≥ 18
NCT03852537	SMART Trial: steroid dosing by biomarker guided titration in critically ill patients with pneumonia	Methylprednisolone. Other: usual care	Methylprednisolone—predetermined dosing table—discontinue if CR < 0.5 mg; 0.5 mg if CRP is 51–100 mmol/L or 0.75 mg/kg if CRP level is 101–150 mmol/L; 1 mg/kg if CRP 151–200 mmol/L or 1.5 mg/kg if CRP level > 200 mmol/L or dose equivalent of oral prednisone for the above	≥ 18
NCT04374071	Early short course corticosteroids in hospitalized patients with COVID-19	Methylprednisolone	1. Methylprednisolone 0.5 to 1 mg/kg/day IV in two divided doses × 3 days 2. Hydroxychloroquine and IV methylprednisolone 0.5 to 1 mg/kg/day in 2 divided doses × 3–7 days	≥ 18

**Fig. 3:**
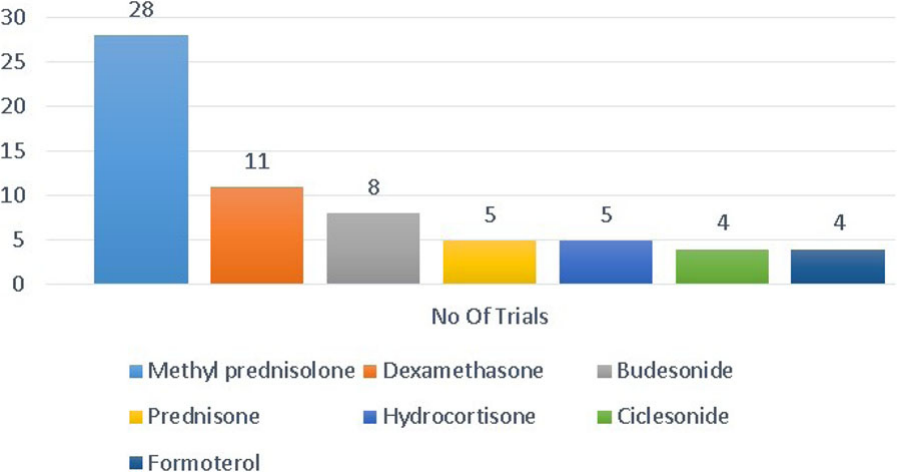
Number of trials using different kinds of steroids

Figure 3 depicts the number of trials studying different types of steroids, showing majority of the trials (*N* = 28) have decided to study the effectiveness of methylprednisolone in the treatment of COVID-19.

### Primary and secondary outcomes

Table 1 summarizes results from all 60 studies. All the trials had clearly defined primary and secondary outcomes of interest, in which only 11 trials had evaluation of respiratory rate as one of their outcomes. Common outcomes measured are respiratory rate, mortality rate, ventilation free days, days in ICU, patient Sequential Organ Failure Assessment (SOFA) score, Murray lung injury score, National Early Warning Score 2 (NEWS2) score, number of patients with treatment failure, rate of remission and progression, blood oxygen saturation, chest x-ray, steroid-related adverse effect, and toxicity monitoring. Table 4 summarizes the consolidation of completed trials with results. All the completed trials have used methylprednisone, dexamethasone, and hydrocortisone as drug of choices.

**Table 4:** Characteristics of published completed trials

Ref	Country	Year of publication	Steroid used	Primary outcomes	***P*** value
[24]	China	2020	Methylprednisolone 396 of 409 [96.8%], dexamethasone 32 of 409 [7.8%] patients—hydrocortisone equivalent	Corticosteroid therapy had higher 28-day mortality rate. Delay in SARS-CoV RNA clearance (*P* = 0.00017)	< 0.05
[25]	USA	2020	Hydrocortisone 200 mg/day and tapered till 50 mg/day	Treatment failure occurred in hydrocortisone patient is 42.1% compared to placebo 50.7%	< 0.045
[26]	Netherlands	2020	Methylprednisolone 80 mg, 250 mg	There was a 79% higher likelihood of two stage improvement in respiratory status	< 0.025
[27]	USA	2020	Hydrocortisone 50 mg, 100 mg	The in-hospital death in treatment group is 30% and 26% compared to no hydrocortisone, i.e. 33%	< 0.05

The data obtained from this review shows that steroids of different doses and types were included in numerous ongoing clinical trials. Their safety and efficacy in managing symptoms of COVID-19, especially in the pneumonia stage, were tested. The trials also included patients of different age groups at different stages of COVID-19. The COVID-19 infection goes through three stages from asymptomatic phase to ARDS (acute respiratory distress syndrome) phase. The 2019-nCoV, after entering the nasal cavity, adheres to the epithelial cells and binds to ACE2 receptor [24]. Owing to this reason, it may be evident that different corticosteroids act through different mechanisms to minimize the symptoms of COVID-19 infection. Table 3 represents the total number of population recruited in each trial, from which we estimate the total ARDS population recruited to be 3880 patients with disease stages ranging from moderate to severe respiratory distress of which methylprednisolone was the most commonly used corticosteroids. A study by H.P. Wiedemann et al. showed that methylprednisolone increased mortality rates by at least 14 days after the onset of ARDS, which gives an impression that the routine use of methylprednisolone is not effective in ARDS [25]. Another study by Nelson Lee et al. shows that SARS-CoV RNA concentrations in the second and third week of illness were significantly higher in patients who received early hydrocortisone treatment compared to placebo; thus, it is recommended to be avoided, but can be cautiously used in SARS [26]. The potential risks associated with high-dose corticosteroids in treating 2019-nCoV pneumonia include secondary infections, long-term complications, and prolonged virus shedding and escalating towards advanced stages [27]. Another study conducted by G.C. Khilnani and H. Vijay registered increased mortality rate (35.7%) with the use of corticosteroids [28–33]. Positively, the RECOVERY trial (Randomised Evaluation of COVID-19 therapy) concluded that in hospitalized patients with COVID-19, corticosteroid reduced 28-day mortality among those receiving invasive mechanical ventilation or oxygen at randomization, but not among patients not receiving respiratory support [34]. Moreover, excessive levels of glucocorticoids have shown to precipitate heart failure by aggravating fluid retention, triggering risk factors like glucose intolerance and dyslipidemia, and by worsening atheromatous vascular disease. Additionally, increased risk of mortality with high serum levels of cortisol have been reported, further establishing a link between use of corticosteroids and increased heart failure risk [35]. Thus, the usage of corticosteroids at various stages of COVID-19 is still questionable with higher mortality rates than the comparator. More information can be gained from results from the completed trials. Though four trials have completed its recruitment, results were not available in the registry. The completed four trials were registered in the Iranian clinical trial registry. The outcomes measured in these trials were mortality rate, need for ICU services, duration of stay in the hospital, assessment of side effects, readmission rate, need for oxygen therapy, blood O_2,_ levels, chest x-ray, PAO2/fio2, and need for invasive mechanical ventilation and intubation.

## Conclusion

Numerous interventional and non-interventional studies are being conducted to study the efficacy of corticosteroids in COVID-19. Corticosteroids can regulate immune-mediated lung injury and decrease the development to respiratory failure and death. Dexamethasone has been reported to reduce the duration of mechanical ventilation. Long-term glucocorticoid therapy has displayed significant improvement in indices of alveolar–capillary membrane permeability and mediators of inflammation and tissue repair. Few preliminary trial findings show promising results and recommend the use of methylprednisolone and dexamethasone in the severe form of the COVID-19. Few studies have reported that early administration of dexamethasone could reduce duration of mechanical ventilation and overall mortality in patients with established moderate to severe ARDS; however, there is insufficient data to prove its benefits over its risk. Routine use of corticosteroids should be favored only after a better insight is obtained, with the completion of these trials.

## Data Availability

The datasets analyzed during the current study will be available from the corresponding author on reasonable request.

## Abbreviations

ACE: Angiotensin-converting enzyme
ARDS: Acute respiratory distress syndrome
ChiCTR: Chinese Clinical Trial Registry
COVID-19: Coronavirus disease 2019
CRiS: Clinical Research Information Service–Republic of Korea
EU: European Union
ICU: Intensive care unit
IRCT: Iranian Registry of Clinical Trials
MERS-CoV: Middle East respiratory syndrome coronavirus
NEWS2: National Early Warning Score 2
RNA: Ribonucleic acid
SARS-CoV: Severe acute respiratory syndrome coronavirus
sCAP: Severe community-acquired pneumonia
SOFA: Sequential Organ Failure Assessment
WHO: World Health Organization
